# Demonstration of Equivalence of Generic Glatiramer Acetate and Copaxone^®^

**DOI:** 10.3389/fphar.2021.760726

**Published:** 2021-12-24

**Authors:** Peter Lipsky, Patrick T. Vallano, Jeffrey Smith, Walter Owens, Daniel Snider, Viswanath Bandaru, Yunfu Sun, Ross Wallingford, Joseph Duncan, Joshua Lewis, Jason Southall, Azeem Ansari, Hong Li

**Affiliations:** ^1^ RILITE Research Institute, Charlottesville, VA, United States; ^2^ Viatris Viatris Research and Development, Morgantown, WV, United States

**Keywords:** glatiramer acetate, copolymer, complex generic, generic equivalence, multiple sclerosis

## Abstract

The objective of the current work was to demonstrate the equivalence of Mylan’s glatiramer acetate (GA) to that of the reference product Copaxone^®^ (COP) using the four criteria for active pharmaceutical ingredient sameness as established by the US Food and Drug Administration (FDA). The reaction scheme used to produce Mylan’s glatiramer acetate (MGA) was compared with that of COP, determined from publicly available literature. Comparative analyses of MGA and COP were performed for physicochemical properties such as amino acid composition and molecular weight distributions. Spectroscopic fingerprints were obtained using circular dichroism spectroscopy. Structural signatures for polymerization and depolymerization including total diethylamine (DEA) content, relative proportions of DEA-adducted amino acids, and N-and C-terminal amino acid sequences were probed with an array of highly sensitive analytical methods. Biological activity of the products was assessed using validated murine Experimental autoimmune encephalomyelitis (EAE) models of multiple sclerosis. MGA is produced using the same fundamental reaction scheme as COP and was shown to have equivalent physicochemical properties and composition. Analyses of multiple structural signatures demonstrated equivalence of MGA and COP with regard to polymerization, depolymerization, and propagational shift. Examination of the impact on prevention and treatment of EAE demonstrated equivalence of MGA and COP with respect to both activity and toxicity, and thereby provided confirmatory evidence of sameness. A rigorous, multi-pronged comparison of MGA and COP produced using an equivalent fundamental reaction scheme demonstrated equivalent physicochemical properties, structural signatures for polymerization and depolymerization, and biological activity as evidenced by comparable effects in EAE. These studies demonstrate the equivalence of MGA and COP, establishing active ingredient sameness by the US Food and Drug Administration (FDA) criteria for GA, and provide compelling evidence that the FDA-approved generic MGA can be substituted for COP for the treatment of patients with relapsing-remitting MS.

## 1 Introduction

Multiple sclerosis (MS) is a chronic inflammatory disease of the central nervous system in which the immune system attacks the protective sheath (myelin) of nerve fibers. Eventually, the disease can cause the nerves to be permanently damaged and result in loss of brain tissue (Joy and Johnston, 2001). It is reported that approximately two million people worldwide are affected by this serious disease, including 400,000 in the United States alone (Browne et al., 2014; Dilokthornsakul et al., 2016). It generally occurs in adults with a peak age of onset of 20–40 years and women are more prone to develop this disease. (Ford et al., 2020). There are various forms of the disease with new symptoms either occurring in isolated attacks (relapsing forms) or building up over time (progressive forms). Currently, the Multiple Sclerosis International Federation describes four types of MS: clinically isolated syndrome (CIS), relapsing-remitting MS (RRMS), primary progressive MS (PPMS), and secondary progressive MS (SPMS) (MS-International-Federation, 2018).

Among the four types of MS, the relapsing–remitting form of MS at onset is the predominant pattern. Approximately 85% of the people develop RRMS at onset and are associated with symptoms and episodes of neurological dysfunction for at least 24 h. About 15% of the people develop the gradually progressive form of the disease from onset (PPMS). Another type of MS is the progressive disease with disability, known as SPMS, which occurs 10–15 years after the onset of the disease. The clinical features of RRMS onset involves the occurrence of unilateral optic neuritis associated with gradual monocular vision loss, pain while moving the eye and altered color vision. (Ford et al., 2020). Other conditions include decreased visual acuity, relative afferent pupillary defect, central scotoma, or impaired color vision, sensory disturbance, bladder dysfunction, cognitive deficits, unilateral painless loss of vision, double vision, limb weakness, ataxia, fatigue, and bowel troubles. (Ford et al., 2020) (Huang et al., 2017)

There is no one particular diagnosis for MS. Diagnosis is based on the clinical episodes and manifestations of neurological dysfunction. It includes a combination of procedures such as patient’ history, physical and clinical examinations, laboratory testing, MRI and other clinical investigations. (Ford et al., 2020).

There is no known cure for MS. Various treatments are available and designed to improve central nervous system function after an attack or prevent subsequent attacks after the initial clinical diagnoses. These disease-modifying therapies (DMTs) have largely an anti-inflammatory effect and are modestly effective at decreasing the number of episodes in RRMS (Vargas and Tyor, 2017). To date there is only one treatment available for the progressive form of MS (Sorensen and Blinkenberg, 2016). In the European countries, two DMTs - delayed release dimethyl fumarate (DMF) and teriflunomide (TRF) - are approved for use as first-line oral therapies. (D’Amico E et al., 2021).

For more than 20 years, Copaxone^®^ (COP; Teva Pharmaceuticals United States Inc, North Wales, PA, United States), has been a first-line treatment for RRMS (Copaxone, 2021). While its mechanisms of action have not been fully elucidated, COP has been shown to exhibit myriad immunological effects (Ruggieri et al., 2007), including binding to major histocompatibility complex proteins to prevent myelin antigen presentation to T-cells (Fridkis-Hareli et al., 1997), and promoting an anti-inflammatory T-cell response (Aharoni et al., 1997; Duda et al., 2000). A daily 20 mg/ml subcutaneous injection of COP has been shown to reduce the frequency of clinical relapses. COP 40 mg, administered three times per week, is also available and shown to significantly reduce underlying disease activity over a 12-month period when compared with placebo (Copaxone, 2021).

Given the utility of COP, generic alternatives are desirable. In the United States, generic medicines are approved through an abbreviated pathway in which the animal and clinical (human) studies that were required of the brand-name medicines to demonstrate safety and effectiveness do not need to be repeated. In order to approve a generic alternative, the US Food and Drug Administration (FDA) requires drug companies to demonstrate that the generic medicine is therapeutically equivalent to the brand-name version of the medicine.

The recently approved Mylan glatiramer acetate (MGA) products were submitted to the FDA through the abbreviated new drug application (ANDA) pathway, one of the various legislative pathways that are available for the approval of pharmaceutical drugs. In general, an ANDA submitted to the FDA must show that the generic medicine is therapeutically equivalent to the brand-name version. This is typically done by providing data within the ANDA that demonstrate the generic medicine to be both pharmaceutically equivalent and bioequivalent to the reference listed drug (RLD) (US Food and Drug Administration, 2018a). Bioequivalence is defined as “the rate and extent of absorption of the test drug do not show a significant difference from the rate and extent of absorption of the RLD when administered at the same molar dose” (US Food and Drug Administration, 2018b). As such, if two drug products are the same dosage form, have the same composition of active and inactive ingredients, and are solution-phase products for administration by injection, they are expected to be absorbed similarly, and are therefore assumed to be bioequivalent (US Food and Drug Administration, 2018c). Accordingly, because MGA is a solution-phase parentally administered drug, bioequivalence to COP is assumed.

The FDA considers drug products to be pharmaceutical equivalents if they contain identical amounts of the same active drug ingredient in the same dosage form and route of administration, and meet compendial or other applicable standards of strength, quality, purity, and identity (US Food and Drug Administration, 2018c). However, demonstrating pharmaceutical equivalence of MGA to COP is nontrivial, as its active ingredient, glatiramer acetate (GA), is not a single molecular entity with a defined structure, but rather a mixture of a multitude of copolymers of various lengths and sequences. Given this complexity, a complete molecular-level analysis of GA is not possible. However, by understanding the linkages between the fundamental chemistry used to synthesize the polymer and the various measurable structural attributes it imparts to the active pharmaceutical ingredient (API), sameness can be established by an appropriate set of highly sensitive analytical tools.

In light of this, in april 2016 the FDA published a list of four criteria that generic sponsors must meet in order to demonstrate API sameness to COP (Copaxone, 2021). These include sameness with respect to fundamental reaction scheme, physicochemical properties, structural signatures for polymerization and depolymerization, and results in a biological assay. The first criterion ensures that the same underlying chemistry is used to synthesize the generic GA and as such provides evidence that the measurable structural signatures imparted by this chemistry are relevant. The second and third criteria assess the sameness of the resulting polymers through the analysis of these measurable structural attributes. The fourth criterion confirms the qualitative and quantitative biochemical sameness established by the first three criteria through the demonstration of equivalence in a biological assay.

On October 3, 2017, the FDA approved MGA 20 and 40 mg/ml products for the treatment of RRMS. The data presented herein demonstrate that MGA meets the FDA’s equivalency criteria, and should therefore be considered a therapeutically equivalent substitute for COP.

## 2 Materials and Methods

### 2.1 Comparison With Fundamental Reaction Scheme of Copaxone®

The synthetic process scheme of MGA was compared with published reaction schemes for GA and patents describing its synthesis (Konfino et al., 2007; Teitelbaum et al., 1971). The amino acids that comprise GA are the levorotatory isomers of glutamic acid, lysine, alanine, and tyrosine.

In the initial step of GA synthesis, alanine, tyrosine, *γ*-O-benzyl glutamic acid, and trifluoroacetyl lysine are converted to reactive N-carboxy anhydrides (NCAs) by treatment with triphosgene. The benzyl and trifluoroacetyl protecting groups are employed to prevent cross-linking of the polymer chains. Next, the NCAs are dissolved in dioxane, after which the polymerization reaction is initiated by the addition of diethylamine (DEA) and allowed to proceed in a highly controlled manner. The resulting crude protected long-chain polymer is then isolated (Intermediate 1). In the subsequent step, Intermediate one is treated with hydrogen bromide in acetic acid under defined time and temperature conditions until the characteristic molecular weight distribution of GA is achieved and the benzyl protecting groups are removed from the glutamic acid side chain. This yields a second isolated intermediate (Intermediate 2), which in a subsequent step is treated with aqueous piperidine to remove the trifluoracetyl protecting groups and purified by diafiltration. Finally, resultant polymer is lyophilized to yield the final drug substance.

### 2.2 Comparative Analysis MGA and Copaxone®

An extensive battery of tests was employed to demonstrate sameness of MGA to COP. Through the course of the development program, numerous individual and orthogonal tests were conducted probing different aspects of the products, including both chemical and biological tests. Here we report on the key tests that followed the FDA’s product-specific guidance on GA (i.e., physicochemical properties, structural signatures for polymerization and depolymerization, and results in a biological assay).

For quantitative analytical methods, equivalence ranges were established based on measured results for COP. For qualitative tests, visual comparisons of spectra or chromatograms were performed. In each of the quantitative analytical tests, a minimum of 20 lots of COP were used to define equivalence ranges. A minimum of three lots of MGA were analyzed in each test and each lot was evaluated individually against the equivalence ranges. For the EAE studies, the MGA lots used were also part of the analytical characterization program. For each EAE study at least 3 separate lots of MGA were included.

A brief description of the analytical methods is as follows. Amino acid analysis was performed using a well-established acid hydrolysis and chemical derivatization methodology. Molecular weight distributions were determined by size-exclusion chromatography. Total DEA was determined by gas chromatography following exhaustive hydrolysis of the polymer, while DEA-adducted amino acids were determined by tandem mass spectrometry. Circular dichroism (CD) analysis was performed by direct analysis on a spectrometer. Terminal sequencing was performed using Edman (N-terminal) or thiocyanate (C-terminal) chemistry with the resultant amino acid derivatives quantified by high-performance liquid chromatography.

The sensitivity of the analytical tests to detect potential differences in glatiramer products was assessed by means of various “negative control” polymers. Because the structural signatures in GA arise based on the fundamental chemistry of the synthetic process, intentional targeted alterations of the process provide a useful means to induce differences between otherwise similar polymers and therefore to test the discriminatory power of the methods.

Mylan’s characterization program included two “Stage 1” negative controls prepared by altering the polymerization reaction. One negative control (NC-1) was prepared using an alternate solvent in place of dioxane, while a second, NC-2, was made by altering the time relationship between monomer addition and initiation of the reaction. Additionally, a “Stage 2” negative control polymer (NC-3) was prepared by using an alternate reagent in place of HBr/HOAc for the partial depolymerization step of the process.

**FIGURE 1:**
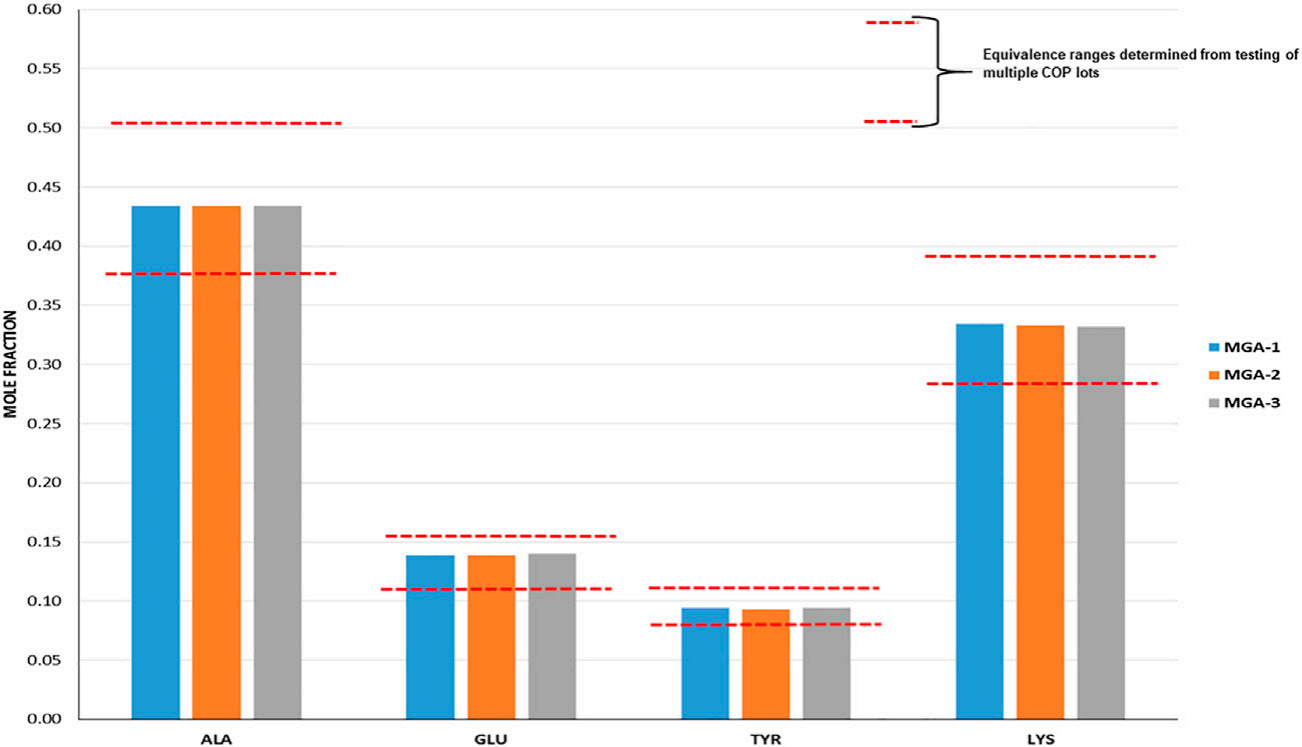
Determination of amino acid composition of three lots of MGA and comparison with COP. The equivalence range for each amino acid determined using multiple lots of COP is indicated by each area defined by parallel dotted red lines.

The intent when preparing the negative controls was to generate polymers that met the COP label claims for molecular weight and amino acid composition (US Food and Drug Administration, 2018d) (17), but that differed with respect to the targeted structural signatures. NC-1, -2, and -3 each met the aforementioned label claims (Mp between 5,000–9,000 Da; amino acid composition within the equivalence ranges shown in Figure 1).

### 2.3 Comparison of MGA and Copaxone® in EAE

Three different Experimental autoimmune encephalomyelitis (EAE) models were used to demonstrate the equivalence *in vivo* of 20 and 40 mg/ml lots of MGA and COP with regard to biological function. In mouse models, development of disease was induced by one of 1) immunization with myelin oligodendrocyte protein (MOG_35-55_), 2) immunization with proteolipid protein (PLP_139-151_), and 3) receipt of encephalitogenic T cells from donor mice immunized with PLP_139-151_. All animal experiments performed in the manuscript were conducted in compliance with institutional guidelines.

#### 2.3.1 MOG35-55-EAE Model

Female C57BL/6 mice randomly were assigned to groups of 12 mice each, and on Day 0 were injected subcutaneously at two sites on the back with complete Freund’s adjuvant (CFA) containing MOG_35-55_ (200 μg/mouse), phosphate buffered saline (PBS), and MGA (500 μg/mouse); and one lot of COP (500 μg/mouse) or vehicle (negative control). Daily, animals were scored for clinical signs of EAE, and body weight was measured.

#### 2.3.2 PLP139-151-EAE Model

The PLP_139-151_-induced EAE preventive model also was employed. Female (SJL/J) mice were randomly assigned to groups of 8–10 mice each. Each mouse was dosed subcutaneously on Day 0 with PLP_139-151_, CFA, PBS, and MGA (500 μg); COP (500 μg); or a negative control group containing no GA. Daily, EAE clinical signs were scored, and body weight recorded.

#### 2.3.3 Adoptive Transfer EAE Model

In the EAE adoptive transfer model, female SJL/J mice were randomly assigned to either a group of 42 donor mice or recipient mice (12 mice/group). Mice in the donor group were immunized by subcutaneous injection of PLP_139-151_ and CFA on Day -13. On Day -3, the spleens and draining lymph nodes were removed, disrupted to prepare individual cells, and pooled. Cells were cultured with PLP_139-151_ to generate encephalitogenic T cells. Cells were then harvested, resuspended in RPMI 1640 medium, counted, and 40 x 10^6^ injected intraperitoneally into naïve-recipient SJL/J mice. Recipient mice were treated daily from Day 0 to Day 9 by subcutaneous injection of MGA (4 mg/dose), COP (4 mg/dose), or a vehicle control. Animals were scored for EAE clinical signs and body weight.

### 2.4 EAE Data Analysis

#### 2.4.1 Calculation of the Percentage Activity

% of sick animals = total number of sick animals ÷ total number in the group x 100

% EAE blocking activity = $\left(1-\frac{\%\;sick\;animal\;in\;test\;group}{\%\;sick\;animal\;in\;animal\;group}\right)\times 100\%\;$

#### 2.4.2 Calculation of the Mean Maximal Score (MMS)

MMS = Σ maximal score of each mouse/number of mice in the group.

#### 2.4.3 Calculation of the Mean Daily Score

Mean daily score = Σ score for the day/number of surviving animals.

## 3.Results and Discussion

### 3.1 Equivalence of Fundamental Reaction Scheme

The fundamental reaction scheme for the synthesis of GA entails the use “activated” forms (N-carboxyanhydrides) of the four constituent amino acids, polymerization in dioxane initiated by DEA, and partial depolymerization in HBr/HOAc (Campos-García et al., 2017). This underlying chemistry gives rise to various structural signatures in the resultant polymer due to, for example, the mechanism of initiation, the relative reactivities of the monomers, and the selectivity of peptide bond scission. MGA is manufactured using this same fundamental reaction scheme and therefore satisfies the first FDA criterion for GA active ingredient sameness.

### 3.2 Equivalence of Physiochemical Properties

Criterion two requires that the physicochemical properties of generic GA, including amino acid composition, molecular weight distribution, and spectroscopic fingerprints, are equivalent to COP.

Amino acid composition is one of the defining characteristics of COP, with the relative amounts of each amino acid listed in the product label **(** Copaxone, 2021 **)**. As part of the sameness assessment, the amino acid compositions of MGA and COP were determined. Results of this analysis, expressed as mole fractions of each amino acid, are presented in Figure 1, and show that all MGA lots fell within the equivalence range.

**FIGURE 2:**
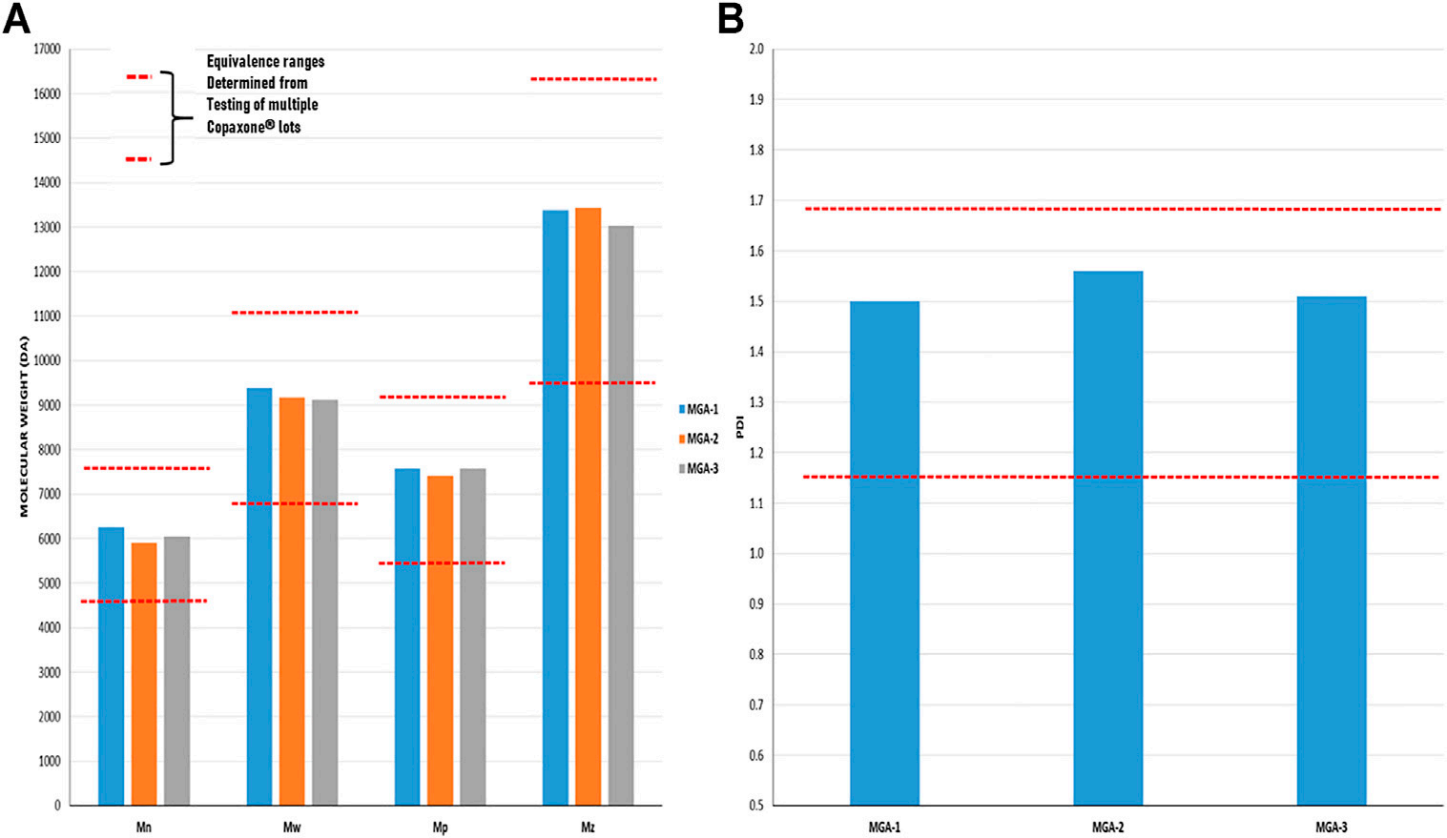
Determination of the molecular weight distribution and Polydispersity Index in three lots of MGA and comparison with COP **(A)** The equivalence range for the molecular weight distribution determined using multiple lots of COP is indicated by each area defined by parallel dotted red lines **(B)** The equivalence range for the polydispersity index determined using multiple lots of COP is indicated by the area defined by parallel dotted red lines.

As with amino acid composition, molecular weight is a defining characteristic of COP with an average value of 5,000–9,000 Da listed in the product label **(** Copaxone, 2021 **)**. In addition to a simple “average” molecular weight analysis, the sameness assessment entailed a full characterization of the molecular weight distributions of the products, an important measure given that GA is a polydisperse polymer. Using size-exclusion chromatography (SEC), the statistical moments of number average, weight average, and Z-average were determined. Additionally, polydispersity index and the molecular weight corresponding to the SEC peak apex (believed to be the molecular weight measure specified in the COP label) were evaluated. Results are provided in Figure 2 and show MGA to be equivalent to COP across each measure.

**FIGURE 3:**
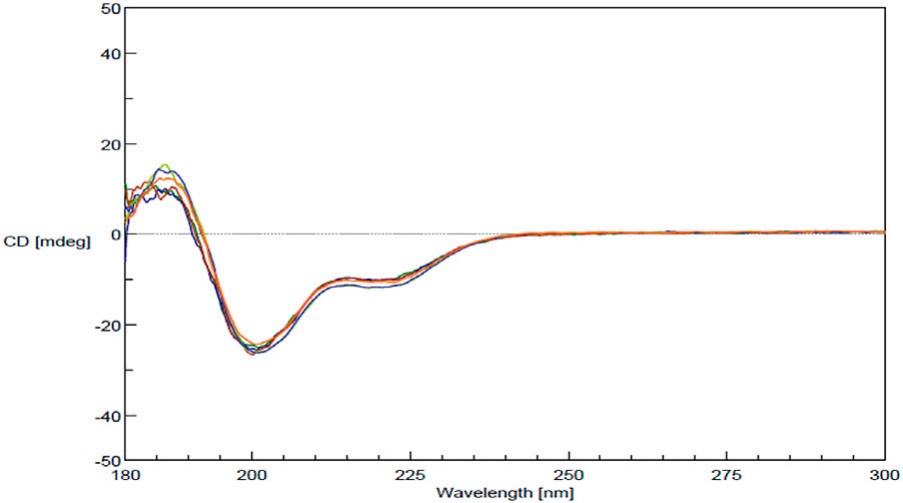
CD spectra for MGA and comparison with COP.

FDA’s physicochemical properties criterion includes a requirement to assess “spectroscopic fingerprints” of the products. The key spectroscopic test used in the characterization of MGA was CD, which was selected based on its sensitivity to detect differences. CD is an optical spectroscopic technique that measures the differential absorption of circularly polarized light in a sample and is commonly used to characterize the secondary structure of proteins (Ranjbar and Gill, 2009). CD spectra obtained for three lots each of MGA and COP are shown in Figure 3. Similar spectral features (e.g., a positive peak around 190 nm and negative peaks around 208 and 222 nm) are seen for all lots of product.

### 3.3 Equivalence of Structural Signatures for Polymerization and Depolymerization

Key structural signatures for polymerization include total DEA and the relative proportions of DEA-adducted amino acids. A well-known initiation mechanism of amine-initiated NCA polymerizations is the normal amine mechanism or (NAM) (Shunmugam, 2017), in which the amine acts as a nucleophile and attacks carbon 5 of the NCA, resulting in ring opening and the generation a new free amino (from the opened NCA) that attacks a second, intact NCA and thereby propagates the reaction. Mechanistic studies on GA polymerization have shown that the reaction proceeds through a NAM pathway (data not shown). One key consequence of this mechanism is that a DEA molecule is incorporated into each polymer chain. Another aspect of this mechanism is that the length of the initial polymer chains (crude, protected polymer at the Intermediate one stage) is affected by the concentration of DEA used in the reaction (higher DEA concentration = shorter chains). Different chain lengths of intermediate polymer can in turn alter the relative incorporation of amino acids into the chains during the propagation step of the reaction, and in turn alter local sequences. Therefore, the total amount of DEA incorporated into the final polymer, a direct reflection of the concentration used in the polymerization step, is a key process signature.

When DEA molecules covalently attach to the NCAs, and ultimately become incorporated into the polymer itself, they link to NCAs of all four amino acids. However, the relative proportions of DEA linkages to these amino acids are nether uniform nor random. Instead, the proportions are determined by the relative rates of reaction of DEA with each NCA, which in turn are a function of the relative concentrations of each NCA and the inherent reactivity (kinetics) of reaction (i.e., some NCAs react faster than others, all other factors equal). As such, the relative proportions of DEA-adducted amino acids serve as another important process signature with which to evaluate MGA and COP.

**FIGURE 4:**
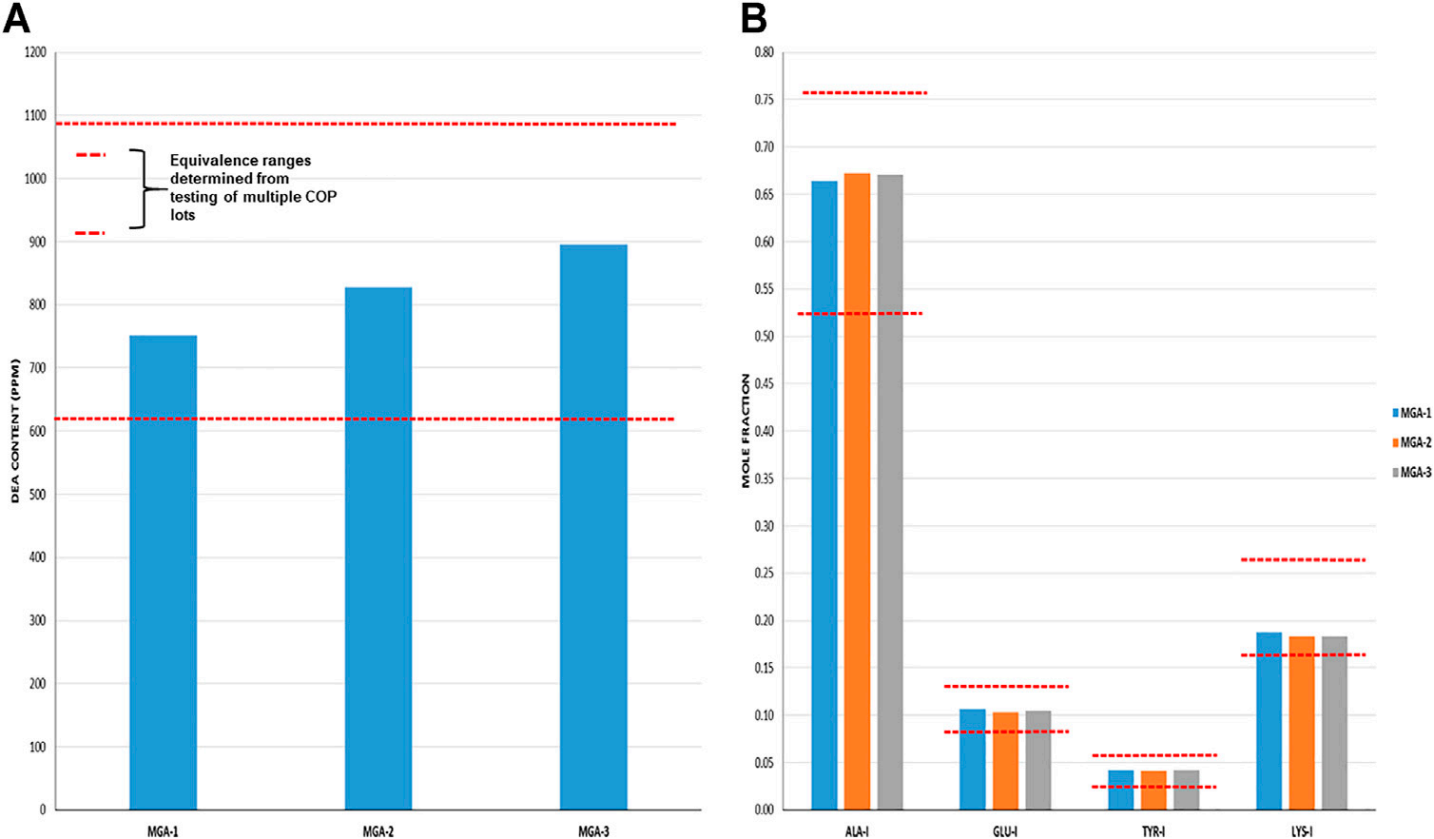
DEA-related analyses of three lots of MGA and comparison with COP **(A)** Total DEA content. The equivalence range is indicated by the area defined by parallel dotted red lines **(B)** Relative amounts of DEA-adducted amino acids. The equivalence ranges are indicated by the area defined by parallel dotted red lines in each graph.

Total DEA determined for MGA and COP is shown in Figure 4A. As can be seen, all MGA lots fell within the equivalence range. DEA-adducted amino acids are presented in Figure 4B and also show equivalence between MGA and COP.

The observed relative proportions of DEA-adducted amino acids reflect the relative reactivities of the monomers, as discussed above, and illustrate the nonrandom aspect of GA synthesis. For example, the mole fraction of alanine-DEA in COP is approximately 0.65, whereas that of tyrosine-DEA is approximately 0.04. These differ markedly from the relative amounts of these amino acids in the bulk COP polymer (approximately 0.43 and 0.09, respectively, as determined from amino acid analysis). These results can be explained by the higher (faster) reactivity of alanine with DEA compared with tyrosine.

Because DEA-related attributes are reflective of the polymerization step of the process, it is not surprising that negative controls NC-1 and NC-2, in which the polymerization process was altered, failed equivalence criteria for at least one DEA test. As shown in Table 1, NC-1 failed for total DEA content and NC-2 failed for DEA-adducted amino acids.

**TABLE 1:** Summary results for negative control samples analyzed in key structural signature tests.

Analytical test	Negative control/Test result		
	Different polymerization solvent (NC1)	Altered time relationship of monomer addition (NC2)	Different depolymerization reagent (NC3)
Total DEA	Fail	Pass	ND
DEA adducts	Pass	Fail	ND
NTS	Fail	Fail	Pass
CTS	Pass	Fail	Fail

Negative controls were tested by the analytical methods listed in table. A “Pass” indicates that all results generated for the analytical tests were within the RLD-based acceptance criteria. A “Fail” indicates that the result did not meet the acceptance criteria. The acceptance criteria range was established by testing multiple lots of RLD, as described in the text. All NC, samples met the RLD, label claims for molecular weight and amino acid composition. Total DEA, and DEA, adducts were not determined (ND) for NC3 because there was no alteration in the polymerization step for this sample.

Amino acid composition at the N- and C-termini was employed as probes for process-related signatures arising from the partial depolymerization step (e.g., preferential sites of peptide bond cleavage), as well as for “propagational shift.” Propagational shift refers to the differential monomer composition along a polymer chain, which in the case of GA, arises due to the differences in rates of amino acid incorporations (due to reaction rate differences similar to that discussed above for DEA). Terminal amino acid composition provides information on propagational shift because the termini that exist in the final product reflect amino acids initially in the interior (both C- and N-termini) as well as on the N-termini of the initial crude protected polymer.

**FIGURE 5:**
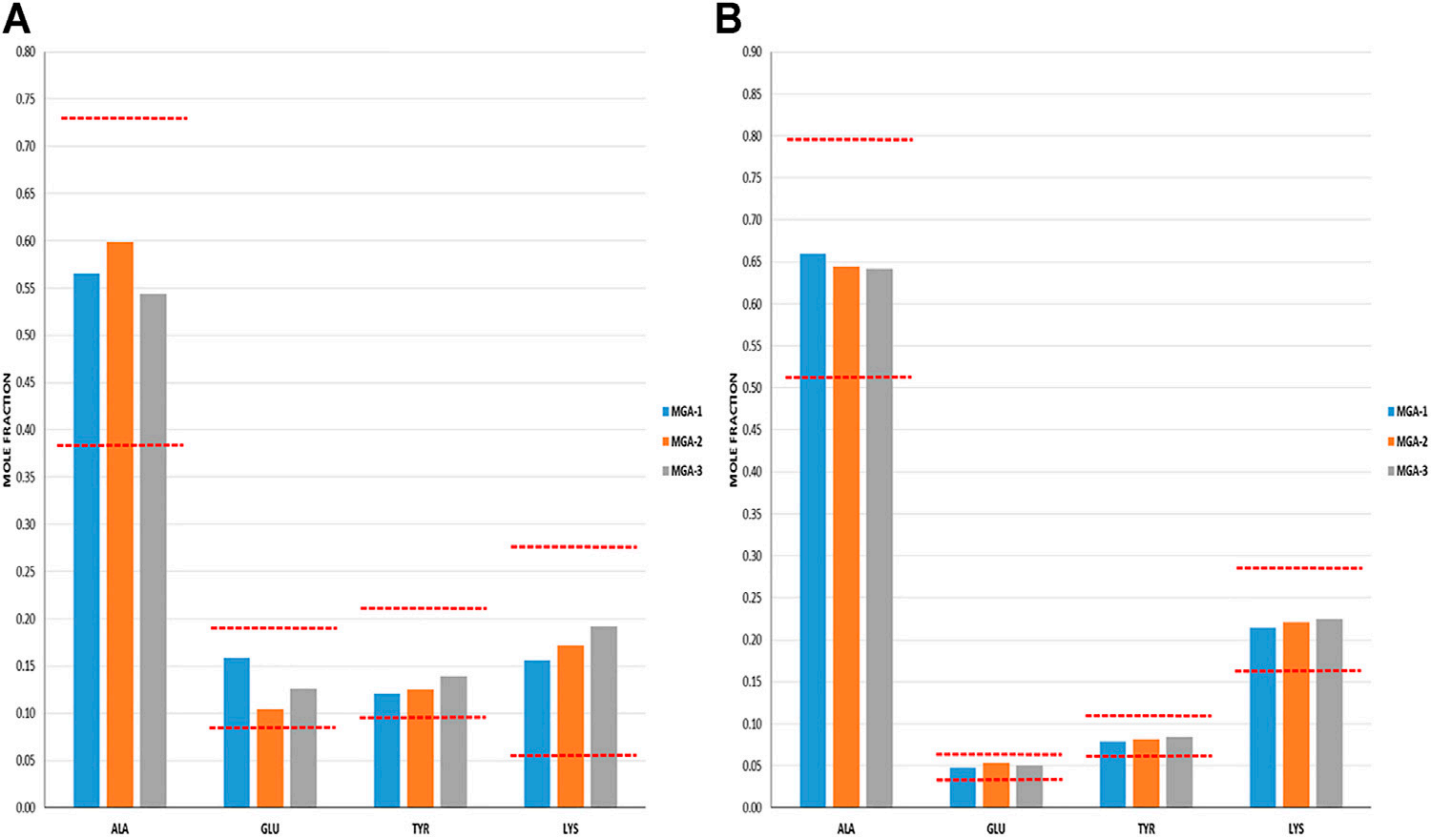
Terminal amino acid composition of three lots of MGA and comparison with COP **(A)** N-terminal amino acid composition. The equivalence range for each terminus determined using multiple lots of COP is indicated by the area defined by parallel dotted red lines **(B)** C-terminal amino acid composition. The equivalence range for each terminus determined using multiple lots of COP is indicated by the area defined by parallel dotted red lines.

Cycle 1 N- and C-terminal amino acid compositions for MGA and COP are shown in Figures 5A, B. In all cases, MGA fell within the equivalence ranges for both terminal sequence analyses.

As was the case with the DEA-adducted amino acids discussed above, the repeatable patterns observed in the relative proportions of amino acids at both the N- and C-termini reflect the nonrandom aspect of GA synthesis and imprinting of process signatures by the underlying chemistry. The prevalence of alanine in excess of its fraction in the bulk polymers (>0.5 vs 0.43) reflects a preference at this amino acid for peptide bond cleavage. In contrast, the abundance of C-terminal glutamic acid is depleted relative to the bulk (≈0.05 vs 0.14) and thus suggests a strong bias away from scission that creates C-terminal glutamic acid residues.

Furthermore, the negative controls were used to demonstrate that terminal amino acid sequencing is sensitive to alterations in propagational shift (polymerization step) as well as depolymerization selectivity. NC-1 and NC-2 were manufactured by altering the polymerization step; however, the depolymerization step was conducted using the standard HBr/HOAc reagent. Tellingly, both of these polymers failed the N-terminal sequencing test and NC-2 failed both the N- and C-terminal tests. This can be explained by alterations in relative incorporation of amino acids that translated into terminal amino acid compositions.

In contrast, polymer NC-3 was made using crude protected polymer (Intermediate 1) manufactured from the standard process and thus had no alteration in propagational shift. The change in the manufacture was in the partial depolymerization step, in which an alternate reagent was used. As seen in Table 1, NC-3 failed the C-terminal sequence test.

These data provide strong evidence that MGA and COP are equivalent in terms of structural signatures for polymerization and partial depolymerization.

### 3.4 Equivalence Within Biological Assays

**FIGURE 6:**
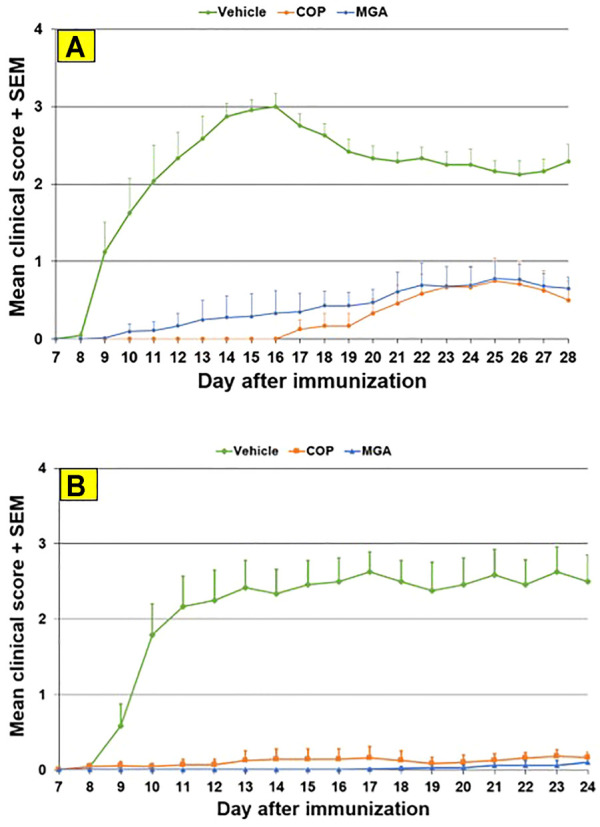
MOG_35-55_-induced EAE: reduction in mean clinical score **(A)** Upper panel shows mean clinical scores following immunization with 20 mg/mL GA lots **(B)** Lower panel shows scores following immunization with 40 mg/mL GA lots. MGA and COP lines show the average scores where multiple lots were tested, as described in the materials and methods.

**FIGURE 7:**
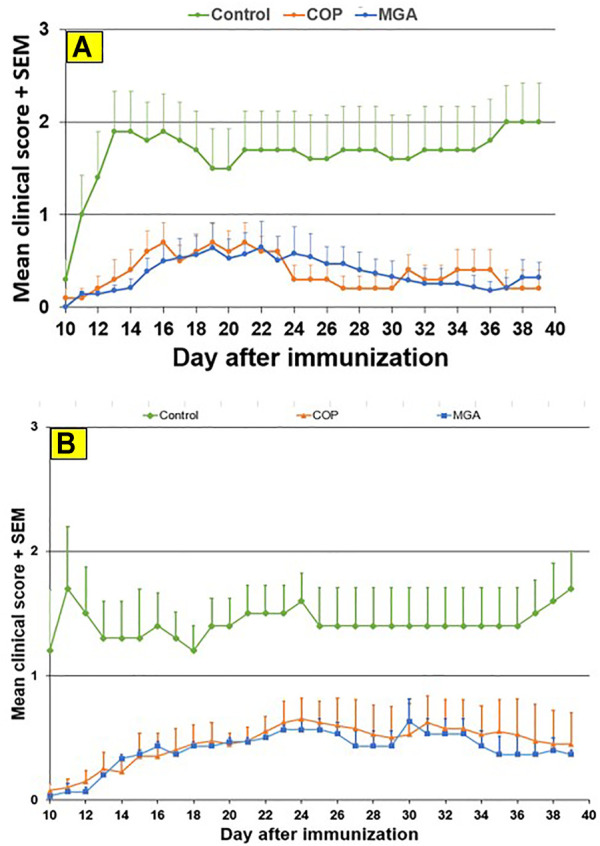
PLP_139-151_-induced EAE: reduction in mean clinical score **(A)** Upper panel shows scores following immunization with 20 mg/mL GA lots **(B)** Lower panel shows scores following immunization with 40 mg/mL GA lots. MGA and COP lines show the average scores where multiple lots were tested, as described in the materials and methods.

**FIGURE 8:**
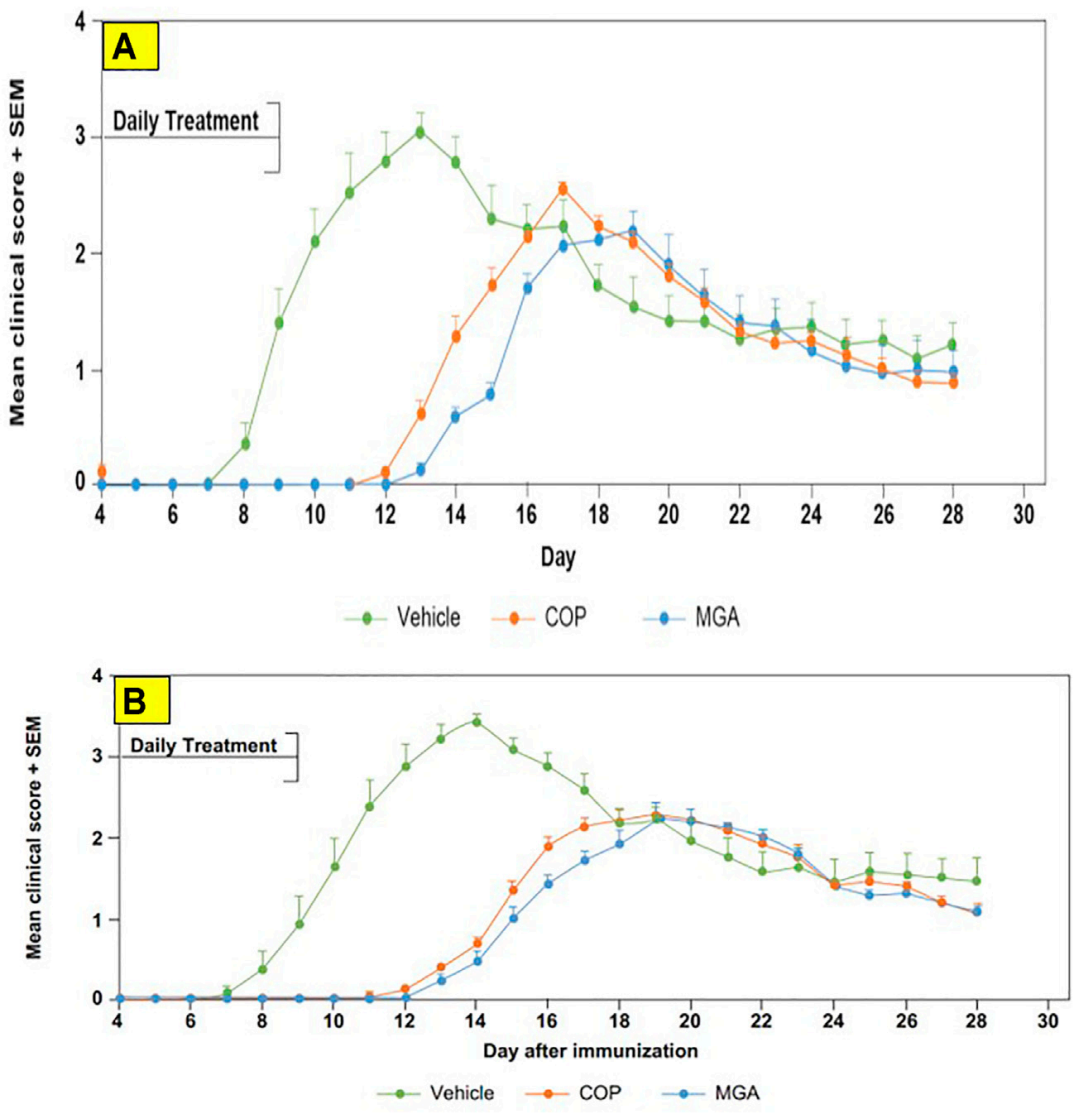
Adoptive transfer EAE: reduction in mean clinical score and time to disease onset **(A)** Upper panel shows scores following immunization of mice with 20 mg/mL GA lots **(B)** Lower panel shows scores following immunization of mice with 40 mg/mL GA lots. MGA and COP treatment of recipients of encephalitogenic T cells from donor mice treated with PLP_139-151_ to induce EAE. As described in the materials and methods, MGA and COP lines show the average scores where multiple lots were tested. GA was administered daily from Day 0 to Day 9. Syringes below the *X*-axes indicate GA administration starting on Day 4 (Days 0–4 not shown).

Biological equivalence was determined by analysis of the impact of COP and MGA in three models of EAE–two preventive models and one therapeutic model. In all models of EAE, MGA and COP demonstrated similar efficacy with respect to their ability to block the induction of disease (Figures 6, 7, 8).

**FIGURE 9:**
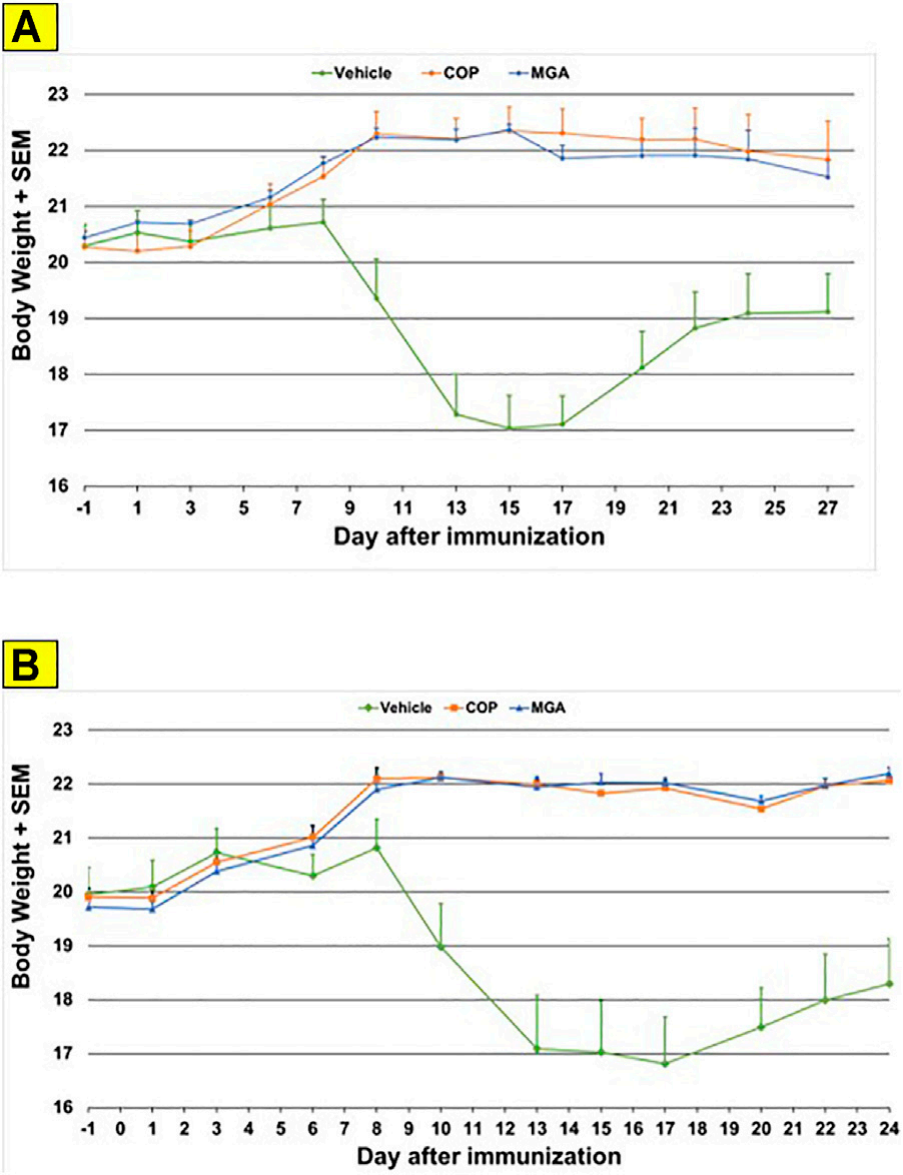
MOG_35-55_-induced EAE: changes in body weight **(A)** Upper panel shows changes in average body weight following immunization with 20 mg/mL GA lots **(B)** Lower panel shows body weight changes following immunization with 40 mg/mL GA lots. These analyses revealed no significant difference between the response to any MGA lot and the COP group response.

In the MOG-EAE induction experiment, treatment with either MGA or COP at 500 μg/mouse substantially blocked EAE induction in mice immunized with MOG_35-55_. Incidence of disease was 100% in control animals and ranged from 17 to 42% in animals treated with 20 mg/ml lots of MGA or COP (Figure 6A, upper panel). Additionally, incidence of disease was 92% in control animals and ranged from 1 to 25% in animals treated with 40 mg/ml lots of MGA or COP (Figure 6B, lower panel). Both COP and MGA significantly and comparably reduced EAE incidence, postponed EAE onset, reduced mean maximum score, and reduced average end score compared with the vehicle group. In addition, all groups treated with MGA or COP had significantly higher average body weight than the vehicle group, which exhibited a decrease in body weight that paralleled disease onset and progression (Figures 9A, B, upper and lower panels, respectively). These analyses revealed no significant difference between the response to any MGA lot and the COP group response.

**FIGURE 10:**
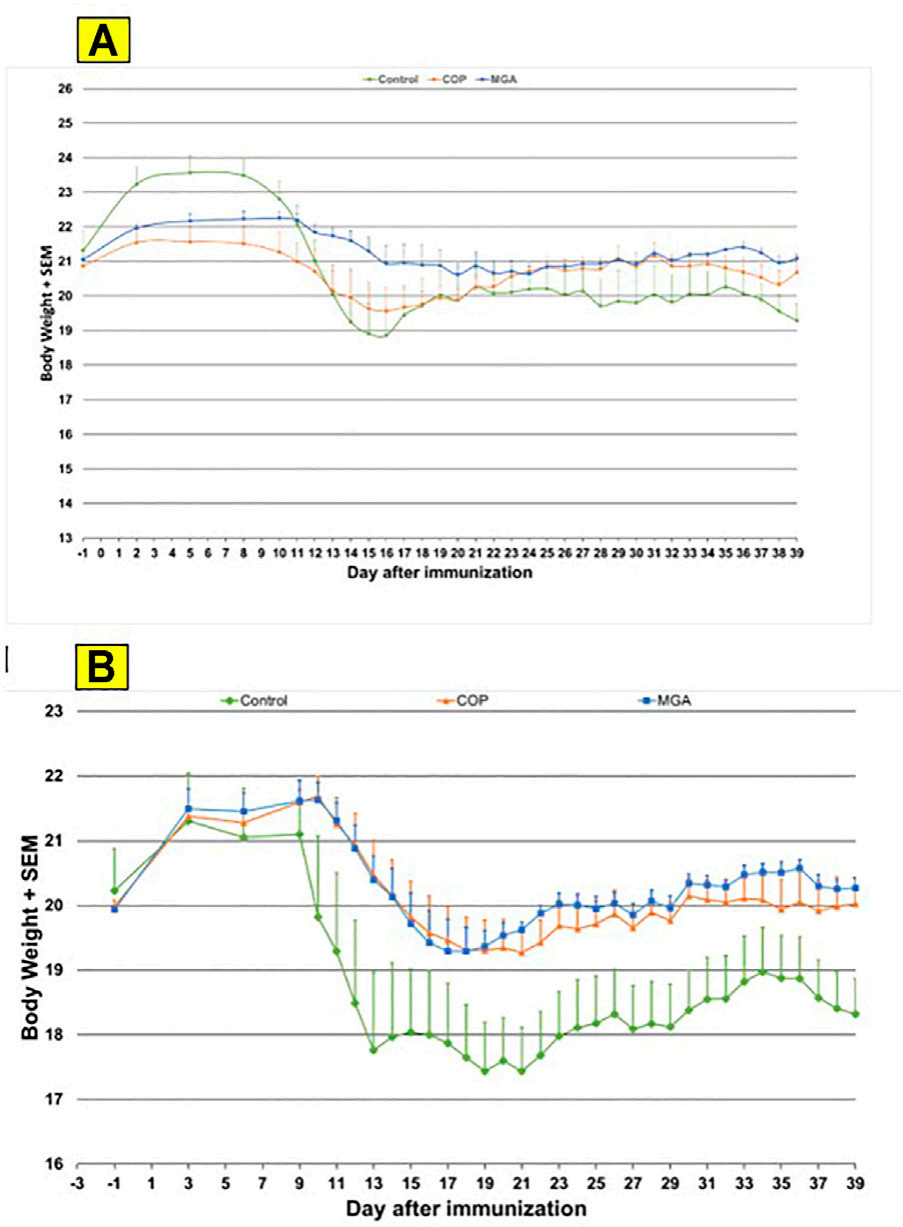
PLP_139-151_-induced EAE: changes in body weight **(A)** Upper panel shows changes in average body weight following immunization with 20 mg/mL GA lots **(B)** Lower panel shows body weight changes following immunization with 40 mg/mL GA lots.

Similar results were obtained in the PLP_139-151_-induced EAE model. Incidence of disease was 100% in control animals. Treatment with 20 or 40 mg/ml MGA or COP lots reduced the severity of the disease relative to the control group (Figures 7A, B **,** upper and lower panels, respectively). A slight decrease in body weight was noted in the control animal group after dosing and appeared to parallel disease onset and progression (Figures 10A, B, upper and lower panels, respectively).

**FIGURE 11:**
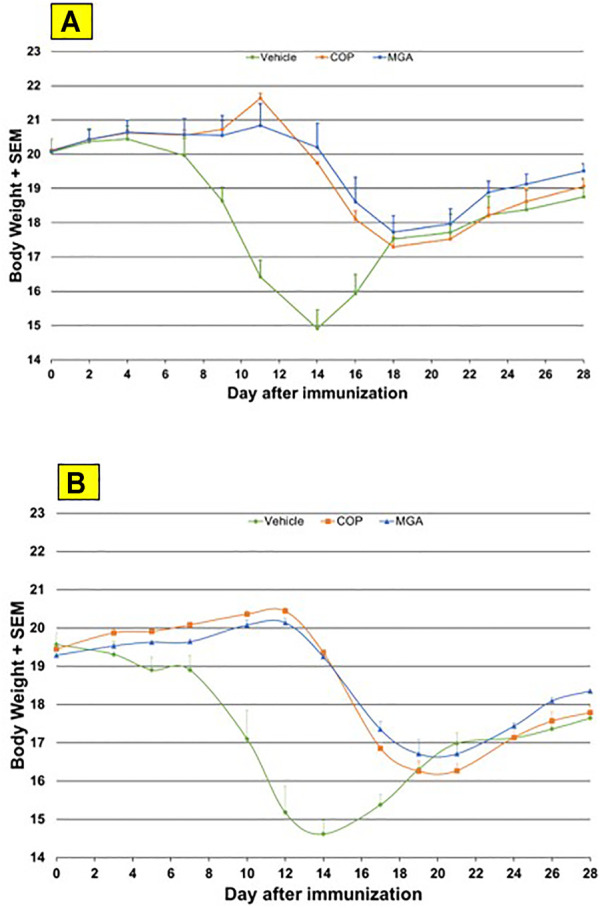
Adoptive transfer EAE: changes in body weight **(A)** Upper panel shows changes in average body weight following immunization of mice with 20 mg/mL GA lots **(B)** Lower panel shows body weight changes following immunization of mice with 40 mg/mL GA lots. MGA and COP treatment of recipients of encephalitogenic T cells from donor mice treated with PLP_139-151_ to induce EAE. As described in the materials and methods, MGA and COP lines show the average scores where multiple lots were tested. GA was administered daily from Day 0 to Day 9. Syringes below the *X*-axes indicate GA administration starting on Day 4 (Days 0–4 not shown).

In the adoptive transfer experiment designed to test the therapeutic effect of test agents after priming of encephalitogenic T cells, MGA and COP suppressed disease comparably. All the tested 20 mg/ml MGA and COP lots postponed EAE onset compared with the vehicle control group, with a median day of onset ranging from Day 13 to Day 15 for treated animals as compared with Day 9 for the vehicle control group (Figure 8A, upper panel). Treatment with 40 mg/ml MGA also suppressed EAE comparably to COP. All the tested MGA and COP lots postponed EAE onset compared with the vehicle control group, with a median day of onset ranging from Day 14 to Day 15.5 as compared with Day 10 for the vehicle control group (Figure 8B **,** lower panel). As anticipated with this model, mice within all groups exhibited weight loss coinciding with the development and progression of disease. (Figures 11A, B, upper and lower panels, respectively).

The economic burden of MS treatment and management is extensive involving various direct and indirect costs, medical and non-medical costs, and informal care costs. The cost of managing MS increases with patients’ age and disease progression. Studies have shown that among the various chronic diseases, MS is the second most cost intensive condition with respect to the direct medical costs incurred. Economic burden includes various costs such as diagnostics costs, physical and clinical examinations, interventions and monitoring, resulting in loss of productivity, employment and quality of life (QoL). Another major burden is the high cost of the disease-modifying therapies (DMTs). (Owens GM et al., 2016) (Garcia Dominguez JM et al., 2019)

Healthcare resource utilization (HCRU) is higher in MS patients compared with patient suffering from other chronic diseases. HCRU such as hospitalization, emergency department visits or frequent visits for physical, speech, or occupational therapy are higher in patients with *de novo* disease. (Owens GM et al., 2016). In 2010, the total lifetime cost per patient for the treatment of MS was estimated to be $4.1 million, of which, 75% of the total HCRU cost was towards treatment with DMTs. Therefore, a careful and delicate balance is required between a robust disease management and value generation when using high-cost DMTs. (Owens GM et al., 2016) (Garcia Dominguez JM et al., 2019)

Treatment and management of MS should be patient-centric and customized as per individual patient’s need. All the resources should be used cautiously. There should be greater collaboration between patients, healthcare providers, managed care organizations and health insurance carriers because many of the available drug therapies are considered therapeutically equivalent by payer pharmacy and therapeutics committees and an individual drug therapy may be determined by pricing contract. It should have plans to improve the clinical outcomes and QoL for patients with MS and thereby minimize the economic burden associated with complex chronic diseases. (Owens GM et al., 2016).

## 4.Conclusion

In 2017, the FDA approved Mylan’s GA products MGA 20 and 40 mg/ml for the treatment of RRMS. Preceding the approval, a series of physicochemical and biological characterization studies was carried out to evaluate the equivalence of MGA to COP under the product-specific guidance developed for GA. The FDA set forth these guidance criteria in 2016 for use by generic sponsors to confirm equivalence and API sameness of generic GA products to COP.

To satisfy these FDA guidance criteria, we compared MGA and COP with regard to fundamental reaction scheme, physicochemical properties, structural signatures for polymerization and partial depolymerization, and equivalence in biological assays, specifically, in each of three different animal models for EAE. In each comparison we established equivalence between MGA and COP.

MGA is manufactured using the same fundamental reaction scheme as that reported for COP. Physicochemical analyses including amino acid composition, molecular weight distribution, and spectroscopic fingerprinting using CD all supported the sameness of MGA and COP. Key process-related structural signatures including total DEA content, the relative amounts of DEA-adducted amino acids, and terminal sequencing at both the carboxyl and amino termini were measured and found to be equivalent in MGA and COP. Polymers manufactured with intentional, targeted alterations further validated the key structural signatures and demonstrated the discriminatory power of the analytical methods.

Three different EAE models were used to compare the biological effects of MGA and COP. The MOG_35-55_ and PLP_130-151_ preventive models were used to represent different forms of human MS, and the PLP_130-151_ therapeutic model was used to represent the effector phase of RRMS (Teitelbaum et al., 1996; Gold et al., 2006; Anderson et al., 2015). We found that MGA blocked the induction of EAE comparably to COP in the two preventive models, and mitigated disease comparably to COP in the therapeutic model.

To summarize, these studies support the equivalency of MGA to COP with regard to structure, chemical properties, and the effects on EAE. These data provide strong evidence that MGA, as an FDA-approved generic, is equivalent to COP for the treatment of RRMS.

## Data Availability

The original contributions presented in the study are included in the article/supplementary material, further inquiries can be directed to the corresponding author.

## Abbreviations

ALA, Alanine; ANDA, Abbreviated New Drug Application; API, Active pharmaceutical ingredient; CD, Circular dichroism; CFA, Complete Freund adjuvant; COP, Copaxone®; CTS, C-terminal sequence; DEA, Diethylamine; EAE, Experimental autoimmune encephalomyelitis; FDA, Food and Drug Administration; GA, Glatiramer acetate; GLU, Glutamic acid; LYS, Lysine; MGA, Mylan’s glatiramer acetate; MMS, Mean maximal score; MN, Number average; MOG, Myelin oligodendrocyte protein; MP, Molecular weight at the peak apex; MS, Multiple sclerosis; MW, Molecular weight; MZ, Z-average; NAM, Normal amine mechanism; NC, Negative control; NCA, N-carboxy anhydride; ND, Not determined; NTS, N-terminal sequence; PBS, Phosphate buffered saline; PLP, Proteolipid protein; RLD, Reference listed drug; RRMS, Relapsing-remitting multiple sclerosis; SEM, Standard error of the mean; TYR, Tyrosine.
